# Case Report: long complete metabolic response assessed by LAFOV FDG-PET/CT to FOLFIRINOX in first-line treatment of metastatic low-differentiated pancreatic carcinoma

**DOI:** 10.3389/fmed.2026.1788442

**Published:** 2026-03-18

**Authors:** Ronan Abgral, Jacques Dzuko Kamga, Jean-philippe Metges, Pierre-Yves Salaun

**Affiliations:** 1 Department of Nuclear Medicine, Brest University Hospital, Brest, France; 2 UMR Inserm 1304 GETBO, Brest, France; 3 Department of Oncology, Brest University Hospital, Brest, France

**Keywords:** Complete response, FDG-PET/CT, FOLFIRINOX, LAFOV, mPDAC

## Abstract

FOLFIRINOX chemotherapy is recommended as first-line treatment for metastatic pancreatic ductal adenocarcinoma (mPDAC). While its efficacy has been well documented in clinical trials, the responses observed have been predominantly partial. To date, only rare cases of complete response (CR) assessed by CT scan have been reported in the literature.

We present a case of sustained metabolic CR over 23 months assessed by LAFOV FDG-PET/CT in a 56-year-old male patient with poorly differentiated mPDAC treated with FOLFIRINOX.

This case highlights the emerging role of latest generation of digital FDG-PET/CT in assessing the therapeutic efficacy of systemic treatments for solid cancers.

## Introduction

Pancreatic ductal adenocarcinoma (PDAC) accounts for approximately 500,000 new cases worldwide and is the 7th leading cause of cancer-related death in both men and women. The prognosis for PDAC remains extremely poor, with only 50% of patients surviving beyond 4 months and a 5-year survival rate of less than 10 % for metastatic disease (1).

FOLFIRINOX (oxaliplatin, irinotecan, fluorouracil, and leucovorin) chemotherapy is recommended as a first-line treatment for metastatic pancreatic ductal adenocarcinoma (mPDAC) (2). The randomized phase III PRODIGE trial showed that FOLFIRINOX significantly improved both median progression-free survival (6.4 vs. 3.3 months; *p* < 0.001) and overall survival (11.1 vs. 6.8 months; *p* < 0.001) compared to gemcitabine in patients with mPDAC. However, of the 54 responding patients in the FOLFIRINOX arm (*n* = 171), only one (0.6%) achieved a complete response (CR) (3).

18F-Fluorodeoxyglucose positron emission tomography/computed tomography (FDG-PET/CT) is an accurate tool for the initial staging of PDAC, particularly for the detection of distant metastases. In addition, FDG-PET/CT is emerging as a useful approach and valuable method for the therapeutic assessment of systemic treatments in metastatic gastrointestinal cancers (4).

## Case Description

**Figure 1:**
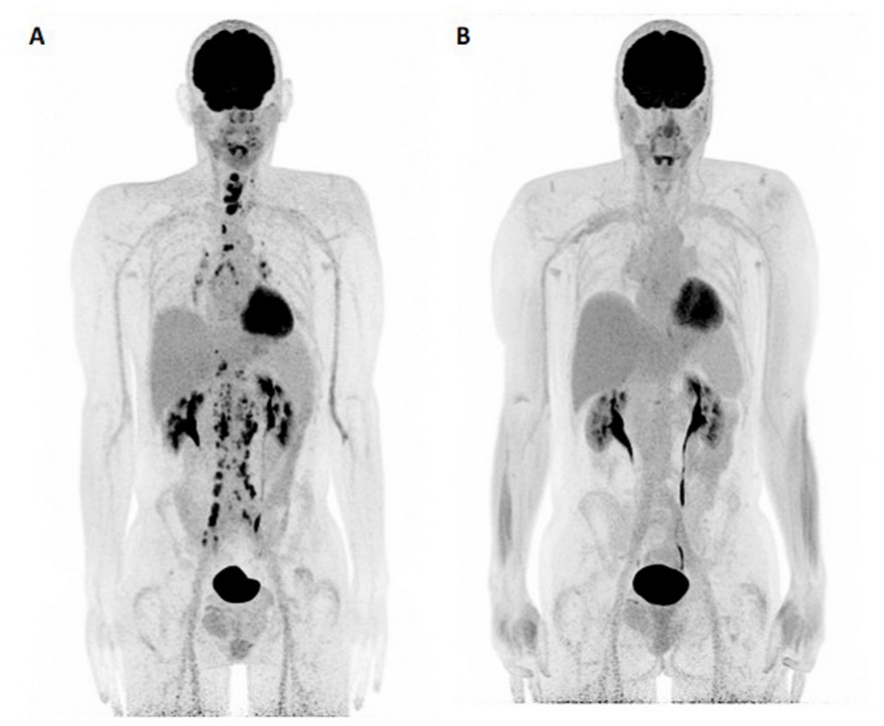
Initial evaluation and follow-up by FDG-PET/CT. **(A, B)** PET MIP showing all lesions at baseline and their disappearance on follow-up.

**Figure 2:**
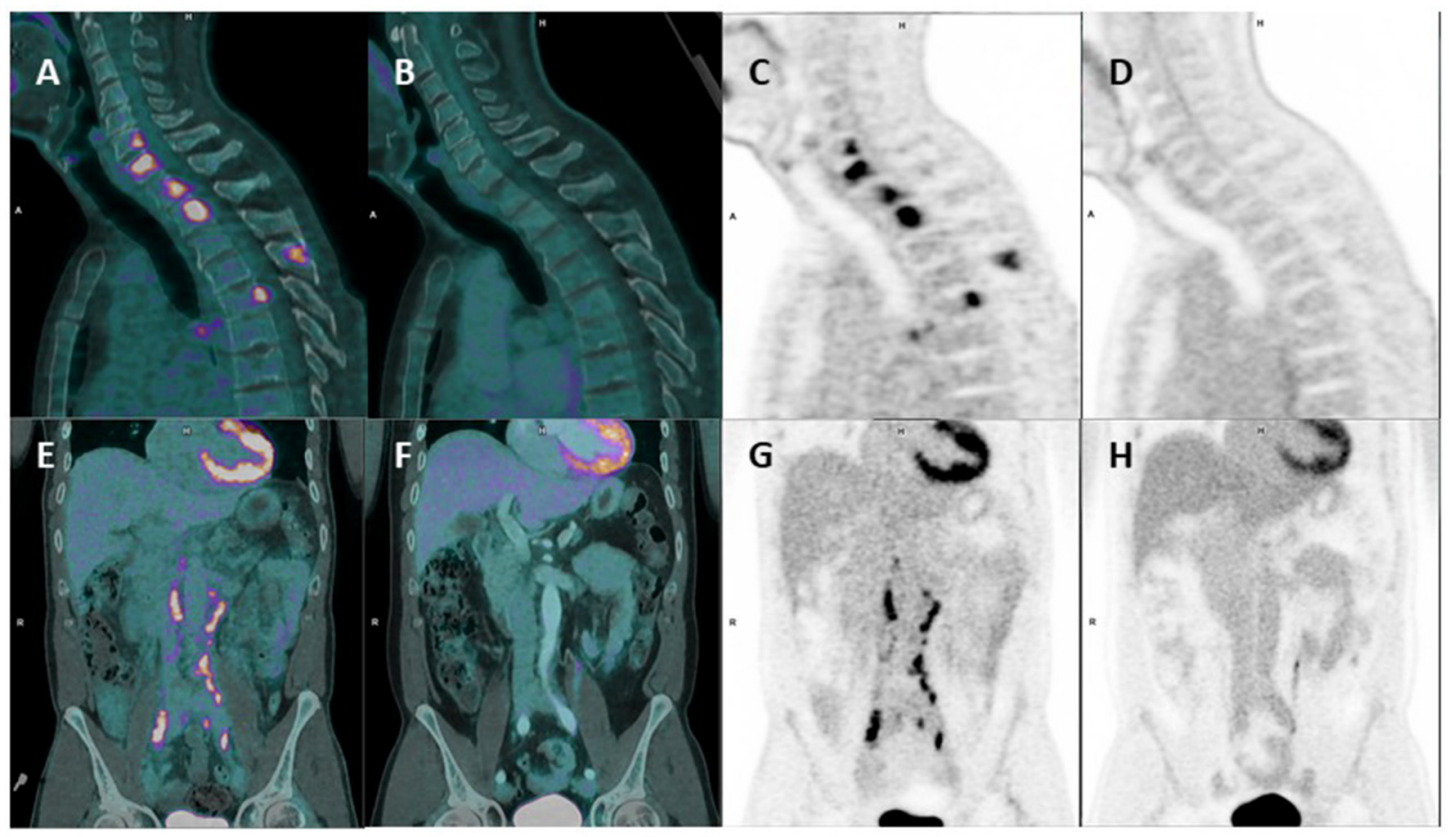
Initial evaluation and follow-up by FDG-PET/CT. **(A, B)** Sagittal PET/CT fusion and PET-only images. **(C, D)** showing the disappearance of cervical and thoracic spinal lesions. **(E, F)** coronal PET/CT fusion and PET-only images. **(G, H)** illustrating the resolution of the para-aortic and common iliac lymph nodes.

We report the case of a 56-year-old man with no prior medical history who was diagnosed with poorly differentiated metastatic pancreatic ductal adenocarcinoma (mPDAC). At diagnosis, the patient was in good general condition (ECOG performance status = 0), with no reported symptoms or abnormal clinical findings. Immunohistochemistry showed positivity for MMR proteins and HER2 negativity, while molecular analysis revealed microsatellite stable (MSS) status and a KRAS mutation, with no BRAF or BRCA mutations. Initial staging with FDG-PET/CT revealed lymph node and bone metastases (Figures 1A, 2A–C, E–G).

According to current guidelines, first-line treatment with FOLFIRINOX was initiated administered at dose of: oxaliplatin 85 mg/m^2^; irinotecan 180 mg/m^2^; leucovorin 400 mg/m^2^; and fluorouracil 400 mg/m^2^ as a bolus, followed by 2,400 mg/m^2^ as a 46-h continuous infusion every 2 weeks. Oxaliplatin was discontinued from the 5th cycle onward due to grade 2 hepatic cytolysis associated with grade 1 peripheral neuropathy. Following improvement in liver function tests and stabilization of neuropathy, oxaliplatin was reintroduced at the 8th cycle with a 20% dose reduction. However, it was permanently discontinued at the 10th cycle due to recurrence of hepatic cytolysis and peripheral neuropathy. Neuropathy subsequently remained stable at grade 1, and hepatic cytolysis completely resolved. Irinotecan was omitted at the 26th cycle due to esophagitis, and its dose was subsequently reduced by 17% for treatment continuation. Other adverse events were mainly gastrointestinal, consisting of nausea and vomiting, particularly at treatment initiation.

**Figure 3:**
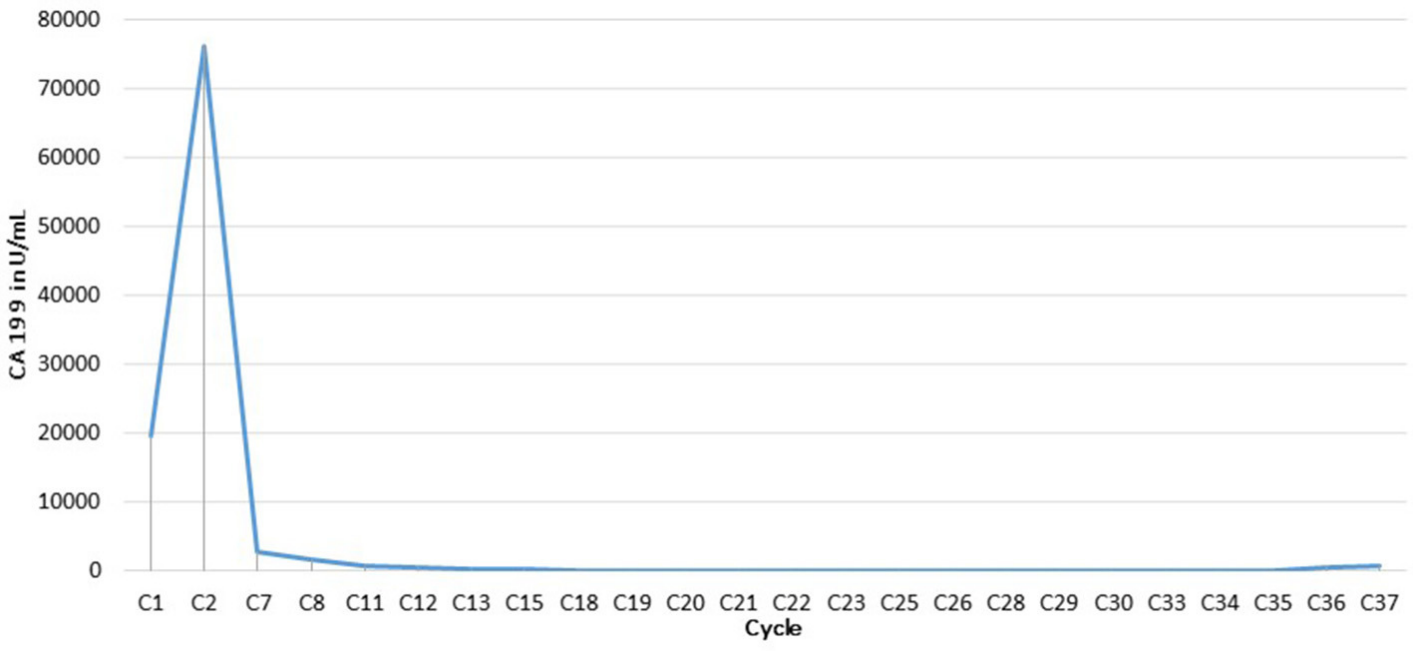
CA 19-9 (U/ml) changes over the course of chemotherapy.

Follow-up FDG-PET/CT demonstrated a complete metabolic response (CMR) of the target lesions according to PERCIST criteria (Figures 1B, 2B–D, F–H). This response was correlated with a marked decrease in CA 19-9 levels, from more than 75,000 U/mL to within the normal range (Figure 3). Remission has been maintained for more than 23 months. The patient has remained in excellent general condition with an ECOG performance status of 0, continuing all usual daily activities. The baseline examination was performed using a conventional digital PET/CT without iodinated contrast enhancement, whereas response assessment was conducted using a long axial field-of-view (LAFOV) PET/CT with contrast-enhanced CT acquired during the portal venous phase.

## Discussion

Platinum-based chemotherapy has been associated with improved overall survival in patients with pancreatic adenocarcinoma harboring germline BRCA1 or BRCA2 mutations (5–7). This was not the case for our patient, in whom no BRCA mutation was identified. Moreover, the detected KRAS mutation is not known to confer specific sensitivity to FOLFIRINOX or FOLFIRI according to available data (8). Historically, gemcitabine monotherapy was the standard treatment for unresectable or recurrent disease (9). Subsequently, phase III trials demonstrated the superiority of FOLFIRINOX and gemcitabine plus nab-paclitaxel over gemcitabine alone (3, 10).

To our knowledge, this represents one of the rare cases of CMR observed on FDG-PET/CT in a patient with mPDAC treated with FOLFIRINOX. To date, only four cases of complete response assessed by CT have been reported; among them, one patient had a FDG-PET/CT demonstrating a CMR (11–14).

Therapeutic assessment was performed using a latest-generation LAFOV PET system, approximately ten-fold more sensitive compared with the digital PET system used at baseline, thereby strengthening the validity of this complete response. Although two different imaging systems were used, the sequence of their use limits the potential impact of this methodological heterogeneity. The conventional digital PET performed at diagnosis, being less sensitive, may theoretically have failed to detect very small additional lesions; however, this would not have altered the already metastatic stage of the disease. Conversely, the LAFOV PET/CT used for therapeutic evaluation provides an excellent negative predictive value. Therefore, the demonstration of a CMR with this system strongly suggests that it would also have been observed with a conventional PET system. Furthermore, iodinated contrast enhancement does not influence metabolic response assessment. The literature indicates that CMR assessed by functional imaging often precedes morphological response in metastatic gastrointestinal cancers (15, 16). A literature review of previously published cases of complete response in mPDAC treated with FOLFIRINOX is summarized in Table 1 (11–14).

**Table 1:** Summary of previously reported cases of complete response in mPDAC treated with FOLFIRINOX.

**Ref.**	**Age/sex**	**Metastatic sites**	**Differentiation**	**Molecular profile**	**Chemotherapy**	**Type of CR**	**Initial clinical signs**	**Toxicities/ adaptations**	**Initial CA 19-9**	**CA 19-9 at CR**	**Imaging**	**CR duration**
Our case	56/M	Lymph nodes, bone	Poorly differentiated	MMR positivity and HER2 negativity; MSS and KRAS mutation; no BRAF or BRCA mutations	FOLFIRINOX → FOLFIRI	Radiological CR	General condition alteration, PS 0–1	Neuropathy → oxaliplatin discontinued; Esophagitis → irinotecan dose reduction	7,5000 U/mL	Normalized	PET-CT	23 months
Shelemey 2020 (PMID: 34031062)	59/F	Liver	Moderately differentiated	MSI-stable; CCND1 amplification; KRAS (G12D) and TP53 (G245S) mutations; BRCA1/2–, PALB2–	FOLFIRINOX → FOLFIRI	Radiological CR	Pain, nausea, sweating; PS 1	Neuropathy → oxaliplatin discontinued	↑ 14514 U/mL; peak at 35,170 U/mL	Normalized	CT/MRI	>4 years
Tsujie 2020 (PMID: 32698268)	46/F	Distant lymph nodes	ND	BRCA1/2 negative	FOLFIRINOX → FOLFIRI	Histological and radiological CR	Obstructive jaundice	Neuropathy → oxaliplatin discontinued	↑ 71795.1 U/mL	Normalized	CT/PET-CT	≥4 years
Nikolaou 2015 (PMID: 26090249)	51/M	Post-surgery: lymph nodes, liver, lungs	ND	ND	FOLFIRINOX → FOLFIRI	Radiological CR	Abdominal pain; jaundice; weight loss; PS 0	Neuropathy → oxaliplatin discontinued; Hematologic toxicity; Digestive toxicity with steatohepatitis	↑ 12000 U/mL	Normalized	CT	≥3 years
Yildirim 2023 (PMID: 36729128)	42/F	Liver	ND	BRCA2 mutated	FOLFIRINOX	Radiological CR	ND	ND	ND	ND	CT	>5 years

CR, complete response; MMR, mismatch repair proteins; HER2: human epidermal growth factor receptor 2; MSS: microsatellite stable; MSI: microsatellite instability; PS: performance status; CT: computed tomography; MRI: magnetic resonance imaging; PET-CT: positron emission tomography-computed tomography; ND: Not described; CA 19-9: carbohydrate antigen 19-9; BRCA1/2: breast cancer gene ½; PALB2, partner and localizer of BRCA2; CCND1: cyclin D1 gene; TP53: tumor protein p53 gene.

## Conclusion

We report a case of complete response of a metastatic PDAC on FDG-PET/CT using a more sensitive LAFOV system, highlighting the potential of FDG-PET/CT for therapeutic assessment of solid cancers in future clinical trials, particularly with these highly sensitive innovative systems.

## Data Availability

The original contributions presented in the study are included in the article/supplementary material, further inquiries can be directed to the corresponding author.

## References

[1] Sung H Ferlay J Siegel RL Laversanne M Soerjomataram I Jemal A . Global Cancer statistics 2020: GLOBOCAN estimates of incidence and mortality worldwide for 36 cancers in 185 countries. CA Cancer J Clin. (2021) 71:209–49. doi: 10.3322/caac.21660 33538338

[2] Tempero MA Malafa MP Al-Hawary M Behrman SW Benson AB Cardin DB . Pancreatic adenocarcinoma, version 2.2021, NCCN clinical practice guidelines in oncology. J Natl Compr Cancer Netw. (2021) 19:439–57. doi: 10.6004/jnccn.2021.0017 33845462

[3] Conroy T Desseigne F Ychou M Bouché O Guimbaud R Bécouarn Y . FOLFIRINOX versus gemcitabine for metastatic pancreatic cancer. N Engl J Med. (2011) 364:1817–25. doi: 10.1056/NEJMoa1011923 21561347

[4] Salaün P-Y Abgral R Malard O Querellou-Lefranc S Quere G Wartski M . Good clinical practice recommendations for the use of PET/CT in oncology. Eur J Nucl Med Mol I. (2020) 47:28–50. doi: 10.1007/s00259-019-04553-8 31637482

[5] Kondo T Kanai M Kou T Sakuma T Mochizuki H Kamada M . Association between homologous recombination repair gene mutations and response to oxaliplatin in pancreatic cancer. Oncotarget. (2018) 9:19817–25. doi: 10.18632/oncotarget.24865 29731985 PMC5929428

[6] Blair AB Groot VP Gemenetzis G Wei J Cameron JL Weiss MJ . BRCA1/BRCA2 germline mutation carriers and sporadic pancreatic ductal adenocarcinoma. J Am Coll Surg (2018) 226:630-637.e1. doi: 10.1016/j.jamcollsurg.2017.12.021 29309945 PMC6178809

[7] Golan T Kanji ZS Epelbaum R Devaud N Dagan E Holter S . Overall survival and clinical characteristics of pancreatic cancer in BRCA mutation carriers. Br J Cancer. (2014) 111:1132–8. doi: 10.1038/bjc.2014.418 25072261 PMC4453851

[8] Buscail L Bournet B Cordelier P. Role of oncogenic KRAS in the diagnosis, prognosis and treatment of pancreatic cancer. Nat Rev Gastroenterol Hepatol. (2020) 17:153–68. doi: 10.1038/s41575-019-0245-4 32005945

[9] Burris HA Moore MJ Andersen J Green MR Rothenberg ML Modiano MR . Improvements in survival and clinical benefit with gemcitabine as first-line therapy for patients with advanced pancreas cancer: a randomized trial. J Clin Oncol. (1997) 15:2403–13. doi: 10.1200/JCO.1997.15.6.2403 9196156

[10] Von Hoff DD Ervin T Arena FP Chiorean EG Infante J Moore M . Increased survival in pancreatic cancer with nab-paclitaxel plus gemcitabine. N Engl J Med (2013) 369:1691–1703. doi: 10.1056/NEJMoa1304369 24131140 PMC4631139

[11] Shelemey PT Amaro CP Ng D Falck V Tam VC. Metastatic pancreatic cancer with complete response to FOLFIRINOX treatment. BMJ Case Rep. (2021) 14:e238395. doi: 10.1136/bcr-2020-238395 34031062 PMC8149332

[12] Tsujie M Fumita S Wakasa T Mizuno S Ishikawa H Kitani K . A case of pathological complete response following FOLFIRINOX therapy for pancreatic adenocarcinoma with synchronous distant lymph node metastases. Int J Surg Case Rep. (2020) 72:471–6. doi: 10.1016/j.ijscr.2020.06.044 32698268 PMC7322239

[13] Nikolaou C Matikas A Papavasilopoulou M Mavroudis D Vamvakas L. Prolonged complete response in a patient with metastatic pancreatic adenocarcinoma after FOLFIRINOX chemotherapy and maintenance with FOLFIRI. Case Rep Oncol Med. (2015) 2015:659624. doi: 10.1155/2015/659624 26090249 PMC4454729

[14] Yildirim HC Ismayilov R Akyildiz A Guven DC Abdurrahimli N Dizdar O . A chance in hopeless cancer: 5-year complete remission after oxaliplatin-based therapy in a patient with BRCA2 mutant metastatic pancreatic cancer. Anticancer Drugs. (2023) 34:1190–2. doi: 10.1097/CAD.0000000000001486 36729128

[15] Amrane K Pennec R. le, Schick U, Metges J-P, Abgral R. Complete metabolic response assessed by FDG PET/CT to FOLFOXIRI-bevacizumab in first-line treatment of brafv600e mutated metastatic colorectal cancer. Clin Nucl Med. (2020)45:707–8. 32657876 10.1097/RLU.0000000000003190

[16] Amrane K Ollivier L Salaun P-Y Metges J-P Abgral R. Complete metabolic response assessed by FDG PET/CT to FOLFIRI-aflibercept in second-line treatment of metastatic colorectal cancer. Clin Nucl Med. (2019) 44:578–9. 31107745 10.1097/RLU.0000000000002611

